# Examining appropriate diagnosis and treatment of malaria: availability and use of rapid diagnostic tests and artemisinin-based combination therapy in public and private health facilities in south east Nigeria

**DOI:** 10.1186/1471-2458-10-486

**Published:** 2010-08-16

**Authors:** Benjamin SC Uzochukwu, Lausdeus O Chiegboka, Chibuike Enwereuzo, Usonwanne Nwosu, David Okorafor, Obinna E Onwujekwe, Nkoli P Uguru, Florence T Sibeudu, Ogochukwu P Ezeoke

**Affiliations:** 1 Department of Community Medicine, College of Medicine, University of Nigeria, Enugu-campus, Nigeria; 2 Health Policy Research Group, College of Medicine, University of Nigeria, Enugu-campus, Nigeria; 3 Department of Health Administration and Management, College of Medicine, University of Nigeria, Enugu-campus, Nigeria; 4 Department of Nursing Sciences, Nnamdi Azikiwe University, Awka Nigeria

## Abstract

**Background:**

Rapid diagnostic tests (RDTs) and Artemisinin-based combination therapy (ACT) have been widely advocated by government and the international community as cost-effective tools for diagnosis and treatment of malaria. ACTs are now the first line treatment drug for malaria in Nigeria and RDTs have been introduced by the government to bridge the existing gaps in proper diagnosis. However, it is not known how readily available these RDTs and ACTs are in public and private health facilities and whether health workers are actually using them. Hence, this study investigated the levels of availability and use of RDTs and ACTs in these facilities.

**Methods:**

The study was undertaken in Enugu state, southeast Nigeria in March 2009. Data was collected from heads of 74 public and private health facilities on the availability and use of RDTs and ACTs. Also, the availability of RDTs and the types of ACTs that were available in the facilities were documented.

**Results:**

Only 31.1% of the health facilities used RDTs to diagnose malaria. The majority used the syndromic approach. However, 61.1% of healthcare providers were aware of RDTs. RDTs were available in 53.3% of the facilities. Public health facilities and health facilities in the urban areas were using RDTs more and these were mainly bought from pharmacy shops and supplied by NGOs. The main reasons given for non use are unreliability of RDTs, supply issues, costs, preference for other methods of diagnosis and providers' ignorance. ACTs were the drug of choice for most public health facilities and the drugs were readily available in these facilities.

**Conclusion:**

Although many providers were knowledgeable about RDTs, not many facilities used it. ACTS were readily available and used in public but not private health facilities. However, the reported use of ACTs with limited proper diagnosis implies that there could be high incidence of inappropriate case management of malaria which can also increase the economic burden of illnesses. Government and donors should ensure constant availability of RDTs in both public and private facilities, so that every treatment with ACTs is accompanied with proper diagnosis.

## Background

Malaria remains a public health problem. Worldwide, it is estimated that around 350-500 million clinical malaria disease episodes occur annually [1]. Estimates show that nearly 60% of the cases of clinical malaria and 700,000 to 1.3 million deaths attributable to malaria (over 90%) occur in sub-Saharan Africa [1]. Nigeria is known for high prevalence of malaria [2,3] and accounts for a quarter of all malaria cases in the WHO African region [4]. It is a leading cause of morbidity and mortality in the country [3]. Available records show that at least 50% of the population of the country suffers from at least one episode of clinical malaria each year and it accounts for over 45% of outpatient visits, 25 and 30% of infant and childhood deaths, respectively and 11% of maternal mortality [3]. In addition, about 12% of gross domestic product is lost to malaria in Nigeria [5].

Prompt and accurate diagnosis of malaria is part of effective disease management and the diagnostic approaches most commonly used are based on the symptoms and signs of the disease and microscopic diagnosis. All these methods have their disadvantages [6-8] which have favoured the introduction and use of rapid diagnostic tests (RDTs).

In Nigeria, Parasight TML ICT and OptiMAL became commercially available in 2000. More recently, others like SD Biolin were also introduced into the Nigerian market. Studies to evaluate the efficacy of these RDTs in Nigeria [9-11] have reported efficacy similar to expert microscopy. In addition, RDTs have been shown to be cost effective in treating malaria in Nigeria [12] and potentially saves the cost and time wasted on presumptive treatment [9].

Presently, over 30 different RDTs brands exist and about 25 million were procured worldwide in 2005 [13]. However, these different brands have different sensitivity and specificity and heat-stability has been a major concern for some especially under field conditions [14]. Also, RDT sensitivity has been shown to depend greatly on user ability to correctly prepare and carry out the test and interpret the results [15,16].

The introduction of RDTs have become a crucial component of malaria control because of the higher-priced artemisinin-based combination therapy (ACT), which was introduced in Nigeria in 2005 as the first-line anti-malarial drug [3], as a result of extensive resistance to chloroquine and sulphadoxine-pyrimethamine (SP). In addition, WHO recommended that combination of antimalarials be used to treat malaria caused by P. falciparum [17], and that laboratory diagnosis be done before patients are treated with ACT [18].

Both RDTs and ACTs therefore are an intrinsic part of the Nigerian malaria control armamentarium. However, the availability of such a test and drug in health care facilities in Nigeria is not known. Hence, it is vital to determine whether both are available and provided by health facilities and to understand constraints in availability and use. This study therefore set out to document the methods of diagnosis of malaria in public and private health facilities and the availability and utilization of RDTs and ACTs in the diagnosis and treatment of malaria in health facilities in Enugu State. The paper contributes to the evidence on the availability and use of RDTs in Nigeria and sub-Saharan Africa

## Methods

### Study Area

The study was carried out in Enugu State in south eastern Nigeria. It has a total of 17 local government areas (LGAs) out of which 4 are urban while 13 are rural. The State operates a district health system (DHS) which is different from what obtains in other parts of the country, with each district serving a population size varying from 160,000-600,000 people [19]. Malaria is holo-endemic in the rural areas and meso-endemic in the urban areas.

### Study Design

This was a descriptive cross sectional study involving public and private healthcare facilities in urban and rural Enugu state. The study was conducted in March 2009

### Sampling

**Figure 1:**
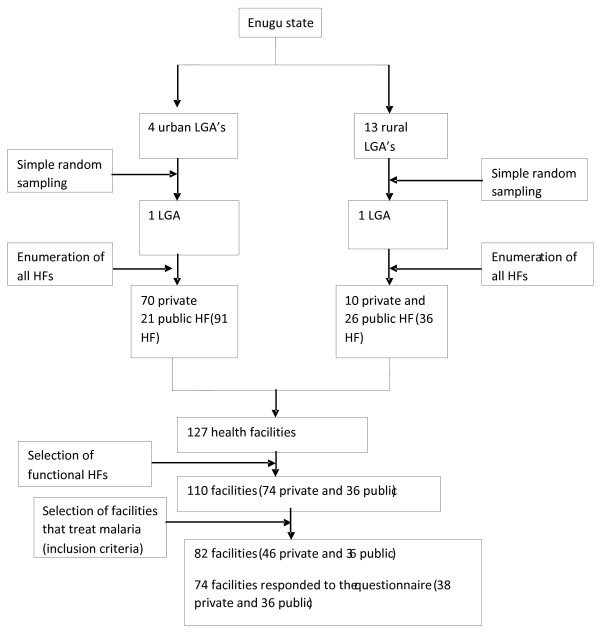
**Flow chart of the sampling method**.

From a sample frame of 4 urban LGAs, Enugu North was chosen by simple random sampling and from a sample frame of 13 rural LGAs, Nkanu East was chosen by simple random sampling. In these two LGAs, all the health facilities, both private and public, were recruited for the study. Overall, there were 21 public health facilities and 70 private health facilities in Enugu North (Urban) giving a total of 91 health facilities. There were 26 public health facilities and 10 private health facilities in Nkanu East (rural) giving a total of 36 health facilities. Of all these only 110 are functional and only 82 facilities treat malaria and were issued questionnaires, while 28 fell into such categories as dental, optic, orthopaedic and other specialized care facilities. Therefore the total number of health facilities issued questionnaires was 82; These were the sample units while the study subjects were the heads of the facilities. The health facilities were stratified into public health facilities (health centers, district hospitals, and teaching hospital) and private health facilities. Figure 1 shows the sampling of the facilities. The average number of health workers treating malaria in the health centers and private clinics is 4, in the secondary health facility it is 10 and 15 in the tertiary hospital. Only one person per health facility was interviewed.

### Data Collection

Using self-administered questionnaires information was collected from heads of 82 health establishments- primary, secondary, tertiary and private- within the area under study. Each questionnaire was made up of 5 sections:

• socio-demographic characterization of the respondents,

• knowledge and utilization of RDTs;

• effectiveness, reliability and comparative accuracy of RDTs (from their experiences with the utilization);

• acquisition/availability supply and economics of RDTs, quality assurance for the RDTs;

• modes by which malaria is diagnosed in the different health facilities, including the costs of the diagnoses.

In addition, using a checklist availability and brand of RDTs and the types of ACTs available in the facilities were noted. The health workers were allowed multiple responses when they were responding to the question on the mode of diagnosis they ever used..

### Data Analysis

The data was analysed for aggregated data from the private and public health facilities using SPSS and EpiInfo software packages. The main influences on availability and use of RDTs and ACTs were evaluated through quantitative analysis of the provider questionnaire responses. As appropriate, chi-square test was used for tests of significance for proportions of categorical variables All tests of significance were done based on a p-level of 0.05.

### Ethical aspects

This research was approved by the College of Medicine, University of Nigeria Enugu-campus Research Ethics Committee. Individual informed consent was obtained from all participants following a verbal and written explanation of study aims and procedures.

## Results

### Characteristics of respondents

Seventy-four facilities correctly filled out and returned their questionnaires given a response rate of 90.2%. More than half 50 (67.6%) of the functional facilities were in the urban area. As shown in table 1, among the doctors and nurses, a majority 22 (95.6%) and 10 (83.3%) respectively were in the urban area, while among the community health extension workers (CHEWs)/community health officers (CHO), a majority 20 (90.9%) were in the rural areas.

**Table 1:** Socio-demographic characteristics of the respondents

Cadre of health worker	Urban N (%)	Rural N (%)	Total N (%)
Doctors	22 (78.3)	1 (21.7)	23 (100)
Nurses	10 (83.3)	2 (16.7)	12 (100)
CHEW/CHO	2 (9.1)	20 (90.9)	22 (100)
Laboratory technologists	16 (94.1)	1 (5.9)	17 (100)
Total	50 (67.6)	24 (32.4)	74 (100)

### Methods of malaria diagnosis

Respondents were asked to state the various methods they used in diagnosing malaria. If they used more than one method, they were asked to say so. As shown in table 2, the most common method for diagnosing malaria in the study area by health workers was syndromic approach, followed by microscopy and then RDT examination. Doctors and laboratory technicians were significantly more likely to use RDTs than CHEWs/CHOs and nurses while the laboratory technicians and nurses were more likely to use microscopy than doctors and CHEWs/CHOs. Also, nurses, CHEW/CHO and doctors were significantly more likely to use syndromic approach than laboratory technicians. Within the groups, all the other health workers were significantly more likely to use syndromic approach except for the laboratory technicians who used microscopy more.

**Table 2:** Diagnostic Methods ever used by healthcare providers

Category of health worker	Types of diagnosis		
	
	RDT N (%)	Microscopy N (%)	Syndromic approach N (%)
Doctor (n - 23)	11 (47.8)	10 (43.5)	18 (78.3)
Nurse (n = 12)	0 (0.0)	7 (58.3)	12 (100)
CHEW/CHO (n = 22)	5 (22.7)	0 (0.0)	20 (90.9)
Laboratory Technician (= 17)	7 (41.2)	12 (70.6)	2 (11.8)
Chi square (p-value)	9.95 (0.019)	23.23 (0.001)	38.12 (0.001)

Total (n = 74)	23 (31.1)	29 (39.2)	52 (70.0)

### Knowledge of RDTs

As shown in table 3, majority of the respondents 45/74 (61.1%) knew about RDTs and most of them learnt it through their co-workers 18 (40%) and conferences 13 (28.9%). Awareness was higher among doctors 18/23 (78.3%) and laboratory technicians 14/17 (82.4%). There was statistically significant differences in awareness among the different cadres of health workers. Also, the proportion of workers aware was higher in the urban area (34/50, 68.0%) than in the rural area (11/24, 45.8%). Health workers in the public facilities were more aware than those in the private facilities and this was statistically significant.

**Table 3:** Awareness of RDTs among respondents

Awareness of RDT	N (%)
**Cadre of health worker **	
Doctors (n = 23)	18 (78.3)
Nurses (n = 12)	3 (25.0)
CHEW/CHO (n = 22)	10 (45.5)
Laboratory technologists (n = 17)	14 (82.4)
Chi-square	14.88
P-value	0.001

**Location**	
Urban (n = 50)	34 (68.0)
Rural (n = 24)	11 (45.8)
Chi-square	3.34
P-value	0.067

**Source of Information (n = 45)**	
Journal	8 (17.8)
Conference	13 (28.9)
Co-worker	18 (40)
Others	6 (13.3)

**By facility**	
Public (n = 36)	26 (72.2)
Private (n = 38)	19 (50.0)
Chi-square	3.83
P-value	0.05

### Availability of RDTs

As shown in table 4, a total of 24 (32.4%) had RDT kits in their health facilities of work at the time of the survey. In the urban area, 13/50 (26.0%) of them and 11/24 (45.8%) of the rural facilities had RDTs, but there was no statistical significant difference (p > 0.05). Among the public health facilities, 14/36 (38.9%) of them and 10/38 (26.3%) of the private facilities had RDTs, but the difference was not significant (p > 0.05). Most of these RDTs were either bought from a pharmacy store 10 (41.7%) or were donated to them by nongovernmental organizations 10 (41.7%). Only 3 (12.5%) of the facilities got their RDTs from the government. However 26 (35.1%) of the respondents knew where to purchase or get RDTs.

**Table 4:** Availability of RDT at Respondents' Place of Work

Variables	N (%)
**RDTs availability at place of work (n = 74)**	24 (32.4)

**Availability in terms of location**	
Urban (n = 50)	13 (26.0)
Rural (n = 24)	11 (45.8)
Chi-square	2.91
P-value	0.088

**Availability in terms of facility **	
Public (n = 36)	14 (38.9)
Private (n = 38)	10 (26.3)
Chi-square	1.33
P-value	0.248

**Sources of RDT (n = 24)**	
Government	3 (12.5)
Bought from a pharmacy	10 (41.7)
Donated by an NGO	10 (41.7)
Not sure	1 (4.1)
**Knowledge of where to get RDT (n = 74)**	26 (35.1)

### Use of RDTs

Table 5 shows that of the 45 that were aware of RDTs, only 23 (51.1%) facilities had actually used it. Most of the users were in urban area 16/34 (47.1%) and were mostly from the public facilities 16/26 (61.5%). Doctors 11/18 (61.1%), laboratory technologists 7/14 (50.0%) and CHEWs/CHOs 5/10 (50.0%) were the main users with no nurse using them. Out of the 23 facilities that had used RDTs only 10 (43.5%) were still using it at the time of the survey. The non users were mainly from the private clinics 8/19 (42.1%) and the reasons given for non use included: unreliability of RDTs, supply issues, cost of the RDTs and preference for other methods of diagnosis.

**Table 5:** Use of RDTs for Diagnosis of Malaria

Variables	N (%)
**Have Ever Used RDT (n = 45)**	
Yes	23 (51.1)
No	22 (48.9)

**Ever used according to location **	
Urban (n = 34)	16 (47.1)
Rural (n = 11)	7 (63.6)
Chi-square	0.91
P-value	0.339

**Ever used RDTs according to type of facility**	
Public (n = 26)	16 (61.5)
Private (n = 19)	7 (36.8)
Chi-square	2.68
P-value	0.102

**Ever used according to professional cadre **	
Doctors (n = 18)	11 (61.1)
Nurses (n = 3)	0 (0.0)
CHEW/CHO (n = 10)	5 (50.0)
Laboratory technologists (n = 14)	7 (50.0)
Chi-square	5.99
P-value	0.102

**Current usage (n = 23)**	
Yes	10 (43.5)
No	13 (56.5)

**Non usage of RDTs according to facility**	
Public (n = 26)	5 (19.2)
Private (n = 19)	8 (42.1)
Chi-square	2.8
P-value	0.09

**Reasons for non use (n = 13)**	
Supply issues	4 (30.8)
Not reliable	8 (61.5)
Prefer other methods	2 (15.4)
Cost of RDT	2 (15.4)

### Perception of usefulness of RDT against other diagnostic methods

Table 6 shows that out of the 23 respondents that had used RDTs, most of them 17 (74%) said RDTs saved time and was better than other diagnostic methods 11 (47.8%), while 4 (17.4%) and 2 (8.7%) of them said RDTs were the same with other diagnostics and were worse than other diagnostic methods respectively. However, 6 (26.1%) were not sure. From the table, supply issues, charge to patients, and ignorance, were the most important limitations of the use of RDTs being 20 (86.9%), 15 (65.2%) and 6 (26.1%) respectively. More than 90% of the respondents rated RDTs to be either good, very good or excellent. A majority of them 16 (69.6%) were satisfied with the benefits of RDT while a few 3 (13%) and 4 (17.4%) were not and indifferent respectively.

**Table 6:** Perception of usefulness of RDT against other diagnostic methods

Variables	N =23 N (%)
**Comparing RDTS with other malarial diagnostic methods**	
RDTS are worse than other diagnostic methods	2 (8.7)
RDTs are same with other diagnostic methods	4 (17.4)
RDT are better than other diagnostic methods	11 (47.8)
Not sure	6 (26.1)
RDT saves time	17 (74)

**Limitations of the use of RDT**	
Charge to patients	15 (65.2)
Needs special skill	2 (8.7)
Supply issue	20 (86.9)
Ignorance on the part of providers	6 (26.1)
Others	1 (4.3)

**Rating of RDT**	
Poor	2 (8.7)
Good	11 (47.8)
Very good	8 (34.8)
Excellent	2 (8.7)

**Benefits of RDT**	
Satisfied	16 (69.6)
Not satisfied	3 (13.0)
Indifferent	4 (17.4)

### Knowledge of effect of temperature and humidity on RDT and RDT preservation at the health facilities

As shown in table 7, of the 23 respondents that had used RDT, 10/16 (62.5%) in public facilities and 6/7 (85.7%) in private facilities said they knew RDT could be affected by temperature. While 6/16 (37.5%) and 2/7 (28.6%) of public and private facilities respondents respectively preserved their RDTs in cold boxes, 4/16 (25.0%) and 2/7 (28.6%) of them in public and private facilities respectively had no special arrangement. There was no statistical significant differences between the private and public facilities in all the variables.

**Table 7:** Respondents' awareness of effect of temperature and humidity on RDT and RDT preservation at the health facilities

Variables	Public N =16 N (%)	Private N =7 N (%)	Chi-square (p-value)
**Aware of Effect of Temperature and Humidity on RDT**	10 (62.5)	6 (85.7)	1.26 (0.26)

**Main method of Preservation of RDT Kits in Health Facilities**	4 (25.0)	2 (28.6)	0.03 (0.617)
No special arrangement	3 (18.8)	1 (14.3)	0.07 (0.648)
Cold boxes	6 (37.5)	2 (28.6)	0.17 (0.532)
Moisture-proof envelopes	0 (0.0)	1 (14.3)	2.39 (0.304)
Others	3 (18.8)	1 (14.3)	0.07 (0.648)

### Drug of choice for treatment of malaria and availability of ACTs in health facilities

Table 8 shows that more of the public health facilities (32, 88.8%) and fewer (13, 34.2%) of the private health facilities reported using ACTs for the treatment of malaria. Private health facilities reported using SP, chloroquine and Artemisinin Monotherapy more than the public health facilities, being SP (12, 31.6%), chloroquine (10, 26.3%) and Artemisinin Monotherapy (3, 7.9%) for private health facilities and SP (2, 5.%6),and chloroquine (2, 5.6%) for public health facilities respectively.. ACTs were available in 32 (88.8%) and 17 (44.7%) of the public and private facilities respectively at the time of this survey (p < 0.05). Most of the public facilities 30 (83.3%) and 14 (36.8%) of the private facilities had Artemether-Lumefantrine (AL). However, Artesunate+Amodiaquine (AA) were found in 13 (36.1%) of the public and 5 (13.2%) of the private health facilities respectively, while Dihydroartemisinin-Piperaquine (DP) were found in 16 (44.4%) of the public and 8 (21.1%) of the private facilities respectively.

**Table 8:** Drug of choice for treatment of malaria and availability of acts in health facilities

Variables	Public N =36 N (%)	Private N =38 N (%)	Chi-square (p-value)
**Drugs used by respondents as first line in the treatment of malaria**			
Artemisinin-Based Combination Therapy	32 (88.8)	13 (34.2)	23.19 (0.0001)
Sulfadoxine-Pyrimethamine (SP)	2 (5.6)	12 (31.6)	8.16 (0.004)
Chloroquine	2 (5.6)	10 (26.3)	5.86 (0.015)
Artemisinin Monotherapy	0 (0.00)	3 (7.9)	2.96 (0.085)

**ACTs available at the time of the survey **	32 (88.8)	17 (44.7)	21.29 (0.0001)
**Types of ACTs seen in the facility **			
Artemether-Lumefantrine (AL)	30 (83.3)	14 (36.8)	16.58 (0.0001)
Artesunate+Amodiaquine (AA)	13 (36.1)	5 (13.2)	5.29 (0.021)
Dihydroartemisinin-Piperaquine (DP)	16 (44.4)	8 (21.1)	4.62 (0.032)

**Various sources of the ACTs in health facilities**			
Government	35 (97.2)	9 (23.7)	41.47 (0.0001)
NGO	2 (5.6)	0 (0.0)	2.17 (0.233)^F^
Purchase from the market	0 (0.0)	26 (68.4)	37.97 (0.0001)
Others	1(2.7)	1(2.6)	0.00 (0.739)^F^

## Discussion

Majority of the facilities still use syndromic approach in the diagnosis of malaria. This finding is not surprising as this had been previously reported from many endemic countries [6]. Using a syndromic approach in diagnosis of malaria means increased likelihood of unnecessary prescribing of antimalarials [11], because some other febrile illnesses that are not malaria might have been treated as malaria. Also the use of, laboratory diagnosis (RDTs and microscopy) was low in this study. The low use of microscopy at 39.2% for diagnosis in this study is similar to the findings of a previous study in Nigeria where a study based on an audit of 665 patients' records from public and private hospitals found that 45% of patients had diagnostic blood slides [20]. Laboratory diagnosis can improve rational provision of malaria treatment service as it has been found that prescribing anti-malarials only after laboratory confirmation reduced the total number of prescriptions by 68% in Malawi [21].

The level of awareness of RDTs by all the providers was not high enough for such an item of enormous utility as RDTs. This calls for the employment of means of creating awareness about RDTs among health workers. If people do not know about a new product and the likely benefits that could accrue from its use, they are not likely to use it and this may lead to market failure. Doctors, CHEWs and laboratory technologists were more likely to be aware of RDTs than nurses. This is not unusual for the laboratory technologists since their main job is to conduct tests. The respondents in urban areas and in public facilities were more likely to be aware of RDTs. The reason for this may be that more attention has been paid to public facilities by government and partner agencies in recent times to improve the case management of malaria in Nigeria.

If more than half of the respondents said RDTs were ever available at their facility of work, and yet the rate of use is low, it then becomes a cause for concern and a threat to the current effort to improve the case management of malaria. Some health workers gave the unreliability of RDTs as a reason for not utilizing available RDT kits. This suggests they do not trust the results despite the fact that RDTs have been found to have a sensitivity of 90.6% and a specificity of 95.9% in Nigeria [9,10]. It has been noted that health workers still treat for malaria even when RDT result is negative [22]. However, most of the health workers who are still using RDTs tend to be satisfied with the results they get. It is possible that poor technique, or even poor preservation of the RDT kits could give rise to poor results which made some health workers to say they stopped using RDTs because it was not reliable. Heat-stability has been noted to be a major concern for some RDTs, especially under field conditions and the health workers may have been exposed to different brands of RDTs including those with health stability problems [14].

Interestingly, RDTs were more available in the rural facilities than urban facilities, a finding that favours the scaling-up of RDTs since a majority of Nigerians live in rural areas. However, the fact that the government was the source of RDTs for only 3 facilities is worrisome. How does W.H.O. intend to promote the RDT use if the government is obviously lacking any interest? This is further confirmed by the fact that the source of information on RDTs was never through formal training sessions promoted by government, as it should be. Although RDTs are still new in Nigeria and currently there are no policies in place on its usage in the diagnosis of malaria except for that from W.H.O, government should play a lead role and make RDTs available to more public facilities.

A majority of the health workers preferred RDTs to blood film microscopy which is a positive finding as this will encourage their use of RDTs. Some of the limitations of the use of RDTs as noted by the health workers included the lack of skills to use it. This is likely to affect the results of RDTs, a fact that has been raised by some authors [23,24]. An intervention area to improve this will be to conduct training and re-training of health workers on the use of RDTs. A positive finding however, is that health workers in both public and private facilities know RDT could be affected by temperature and humidity. This is good for quality control although a good number in the public facilities are not aware of this..

The supply of RDT kits to health facilities in Enugu State has been rather erratic and most RDTs are still purchased in the open market. This could lead to purchase of fake kits if the market is not properly regulated as evidence has shown that the drugs in the Nigerian market may be ineffective, counterfeit or expired [25].

Surprisingly, the drug of choice for the treatment of uncomplicated malaria in the study areas was ACT. This is particularly a positive development in the push to improve the case management of malaria in Nigeria. However, this finding contrasts with a Nigerian survey of malaria control practices that showed that less than a fifth of the primary and secondary health facilities used the recommended ACT [2] and that monotherapies such as Chloroquine, SP, Quinine, Artesunate and Dihydroartemisinin were still widely used for treatment of malaria [26].

ACTs especially the recommended first-line types in the national treatment policy (*Artemether-Lumefantrine (AL) and Artesunate+Amodiaquine (AA) *were readily found in public facilities in diverse trade names. However, it will be noted that as a matter of policy, ACTs are supplied free of charge to children who are under 5 years in public health facilities in Enugu, Nigeria [27] and this may have accounted for the large presence of ACT in these facilities. The Nigerian malaria control programme also delivered 4.5 million courses of ACT in 2006 and 9 million in 2007 [4]. ACTs can also be purchased over the counter without a prescription, and can be dispensed by a non medical personnel. In Nigeria, pregnant women and children receive free SP and ACTs respectively from all public health facilities; however this does not apply to private facilities. In the private sector charges are fixed by the owners of the facilities while in government facilities there are often specified fees for services.

The study shows that ACTs were still not readily available in private facilities but were more available in both types of facilities than RDTs. This calls for strategies to ensure that both ACTs and RDTs are made available to private health care providers at a subsidized rate in form of public private partnership. But in doing this, there is need for sustainable monitoring systems as monitoring and influencing the quality of private services is recognized as a key component of effective malaria treatment [28]

However, the fact that ACTs are readily available (and not RDTs) and are used and considering the fact that most health workers still employ syndromic approach for the diagnosis of malaria, it then means that some patients will be treated with ACTs without laboratory diagnosis. Parasitological diagnosis of malaria is an important parameter leading to the appropriate use of anti-malarial drugs. Improper and abusive use of ACTs without proper diagnosis will have a direct clinical and economic impact [6,29,30]. This therefore calls for interventions at policy and programmatic levels to improve treatment provision. And one obvious intervention will be ensuring that providers stocked adequate doses of RDTs and ACTs and subsequently used them so as to decrease unnecessary treatment and reduce societal costs of malaria.

We did not review the brands of RDTs found in these facilities and we acknowledge this to be a limitation of the study. Further studies should audit the type of RDTs in these facilities as they vary greatly in effectiveness. The sampled facilities may not be a representative sample for the whole country and therefore the results may not be representative of the country. Nevertheless, we believe that this is a representative sample of all health facilities in the area of study and the state and therefore a good starting point in understanding the tremendous gap existing between the optimal, W.H.O. promoted policies of RDTs and ACTs and the real application in practice.

## Conclusion

The knowledge of RDTs among health workers is high, however, it is not readily available. Even among facilities that once used RDT some have stopped using it mostly for the reason of perceived reliability, cost and supply issues. ACTs are readily available in public health facilities but not private health facilities. They are reported to be used for malaria treatment in the study area. Government and donors should therefore ensure the availability of RDTs and ACTs in both public and private facilities. Ensuring the dissemination of information about the existence, usefulness and proven advantages of RDT over other methods of diagnosing malaria will enhance prompt malaria diagnosis and treatment. RDTs should be provided free of charge or at a subsidized rate to health facilities to ensure availability the utilization of RDTs and rational use of ACTs in all health facilities. This will at the long run facilitate reduction in the burden of malaria.

## Competing interests

The authors declare that they have no competing interests.

## Authors' contributions

BSCU, LOC, CE, UN and DO designed performed the field work, analysed and interpreted the results. BSCU wrote the paper. All authors read and approved the final manuscript.

## Pre-publication history

The pre-publication history for this paper can be accessed here:

http://www.biomedcentral.com/1471-2458/10/486/prepub
